# Cryotherapy for the prevention of weekly paclitaxel-induced peripheral adverse events in breast cancer patients

**DOI:** 10.1007/s00520-020-05345-9

**Published:** 2020-02-08

**Authors:** Hideo Shigematsu, Taizo Hirata, Mai Nishina, Daisuke Yasui, Shinji Ozaki

**Affiliations:** 1 Department of Breast Surgery, National Hospital Organization Kure Medical Center and Chugoku Cancer Center, 3-1, Aoyama-cho, Kure City, Hiroshima 737-0023 Japan; 2 Department of Medical Oncology, National Hospital Organization Kure Medical Center and Chugoku Cancer Center, Aoyama-cho, Kure City, Hiroshima Japan; 3 Present Address: Department of Surgical Oncology, Research Institute for Radiation Biology and Medicine, Hiroshima University, 1-2-3 Kasumi, Minami-Ku, Hiroshima City, Hiroshima, 734-8551 Japan; 4 Present Address: Department of Breast Surgery, Hiroshima Prefectural Hospital, Hiroshima, Hiroshima 734-8530 Japan

**Keywords:** Breast cancer, Peripheral neuropathy, Dermatological adverse events, Cryotherapy, Weekly paclitaxel

## Abstract

**Purpose:**

This randomized phase II study was conducted to investigate the efficacy of cryotherapy in preventing peripheral neuropathy and dermatological adverse events in breast cancer patients treated with weekly paclitaxel.

**Methods:**

Patients treated with 12 weekly doses of paclitaxel for breast cancer were randomized (1:1) into a cryotherapy or control group. The primary endpoint was the percentage of patients with a marked decrease in the Functional Assessment of Cancer Therapy-Neurotoxicity (FACT-NTX) score. The secondary endpoints were Patient Neurotoxicity Questionnaire (PNQ), Common Terminology Criteria for Adverse Event (CTCAE) for peripheral neuropathy, and FACT-Taxane score.

**Results:**

Forty-four patients were randomly assigned to the cryotherapy (*n* = 22) or control groups (*n* = 22). The percentage of patients with a marked decrease in FACT-NTX scores was significantly lower in the cryotherapy group than in the control group (41 vs. 73%, *p* = 0.03). The incidence of CTCAE grade ≥ 2 sensory (*p* = 0.001) and motor peripheral neuropathy (*p* = 0.01), and PNQ grade D or higher for sensory peripheral neuropathy (*p* = 0.02), and decrease in the FACT-Taxane score (*p* = 0.02) were also significantly lower in the cryotherapy group than in the control group. There were no serious side effects associated with cryotherapy.

**Conclusion:**

Cryotherapy is an effective approach for prevention of peripheral neuropathy and dermatological adverse events in breast cancer patients treated with weekly paclitaxel.

## Introduction

In a randomized clinical trial evaluating the adjuvant therapy of breast cancer, weekly paclitaxel showed superiority in relapse-free survival and overall survival compared with triweekly paclitaxel [23, 24]. Because weekly paclitaxel regimen results in increased dose intensity and cumulative dosage compared with triweekly paclitaxel, the weekly schedule is associated with a high incidence of peripheral neuropathy [5, 7]. There was a reported incidence of 27 and 8% for grades ≥ 2 and 3/4 peripheral neuropathy with weekly paclitaxel [23]. Although paclitaxel-induced peripheral neuropathy tends to improve after treatment, it may persist in 50–80% of patients, and 25% of these demonstrate strong neuropathy [9, 25]. Another peripheral adverse effect of paclitaxel is dermatological adverse events, which includes nail changes and cutaneous toxicities [12–14]. Systemic investigation showed an incidence of 43.7% for all grade nail changes with paclitaxel treatment [2]. Paclitaxel treatment also causes inflammation of the skin which can lead to cutaneous sclerosis [13, 17]. Although these paclitaxel-related peripheral adverse events are not life threatening, they can substantially deteriorate quality of life (QOL) and subsequently lead to reduction of dose intensity or treatment disruptions [16] [21].

Although there are no established medications for preventing taxane-induced peripheral neuropathy and dermatological adverse events [11], some studies reported that suppression of peripheral circulation via cryotherapy or compression therapy may be effective in preventing these peripheral adverse events. In a retrospective study of breast cancer patients treated with taxane, lower incidences of peripheral neuropathy and nail disorder have been reported in patients treated with cryotherapy than in those who were not [18]. In self-controlled trials of weekly paclitaxel treatment for breast cancer patients, suppression of peripheral circulation via cryotherapy has been reported to be effective in the prevention of peripheral neuropathy [8]. Compression therapy using surgical gloves has also been reported to be useful for the prevention of peripheral neuropathy in self-controlled comparative studies of breast cancer patients treated with nab-paclitaxel every 3 weeks [26]. However, these reports are retrospective studies or self-controlled trials, and thus, the efficacy of cryotherapy for preventing these adverse effects should be confirmed in randomized controlled trials.

With these findings, we conducted a randomized phase II study of cryotherapy for the prevention of peripheral neuropathy and dermatological adverse events in breast cancer patients treated with weekly paclitaxel.

## Methods

### Study design and participants

This randomized phase II controlled trial enrolled breast cancer patients undergoing 12 weekly doses of paclitaxel. The inclusion criteria were a diagnosis of invasive breast cancer, age 20 years or older, scheduled for a regimen of 12 weekly doses of paclitaxel, adequate organ function, an Easter Cooperative Oncology Group Performance Status (PS) of 0 or 1, and no history of taxane-based chemotherapy within the last 12 months. The exclusion criteria were grade ≥ 2 peripheral neuropathy as assessed according to the Common Terminology Criteria for Adverse Event (CTCAE) [1], current treatment with drugs affecting peripheral neuropathy, and comorbidity of Raynaud’s syndrome. Eligible patients were randomized (1:1) into the combination therapy group (cryotherapy using frozen gloves/socks) and the control group. Randomization was performed under a permuted block randomization scheme (block size of four), stratified by age (< 65 vs. 65 and older) and presence of grade1 peripheral neuropathy.

This study adhered to the ethical standards laid down in the Helsinki Declaration and the ethical principles for clinical research. Further, this study was approved by the Ethics Committee of the National Hospital Organization of Kure Medical Center and Chugoku Cancer Center (Approval number 28-70) and is registered in the University Hospital Medical Information Network (UMIN000034966). All patients received a detailed explanation of the study from the primary physician, and written informed consent was obtained prior to enrollment.

### Chemotherapy regimen

Twelve weekly doses of paclitaxel therapy comprised infusion of 80 mg/m^2^ paclitaxel on days 1, 8, and 15 in a 21-day cycle for a total of four cycles. Adjuvant anti-human epidermal growth receptor (HER) therapy was used in HER2-positive breast cancer patients. The addition of bevacizumab was allowed in advanced and recurrent breast cancer.

### Cryotherapy

For cryotherapy, patients wore frozen (− 20 °C) gloves/socks (Elasto-Gel, mittens, and slippers) on both hands and feet continuously for 15 min before paclitaxel infusion until 15 min after infusion in accordance with previous report regarding cryotherapy (total 90 min) [18]. This treatment was performed throughout the 12 weekly doses of paclitaxel in the experimental group. The use of drugs that may affect peripheral neuropathy (e.g., acetyl-l-carnitine, vitamin E, glutamine, gabapentin, tricyclic antidepressants, and opioids) and compression therapy with surgical gloves was prohibited.

### Assessment measures

The primary endpoint was the percentage of patients with a significant decrease in their Functional Assessment of Cancer Therapy-Neurotoxicity (FACT-NTX) score [3]. Briefly, the FACT-NTX is an 11-item tool used for evaluating symptoms and concerns specifically associated with chemotherapy-induced neuropathy. A lower FACT-NTX score means a worsening of peripheral neuropathy. A significant decrease in FACT-NTx score was defined as a decrease exceeding 10% or 6 points of the standard value before weekly paclitaxel [6, 10]. The secondary endpoints were Patient Neurotoxicity Questionnaire (PNQ) grade D or higher [20], Common Terminology Criteria for Adverse Event (CTCAE) grade ≥ 2 for sensory and motor peripheral neuropathy [1], Functional Assessment of Cancer Therapy-Taxane (FACT-Taxane) score [3], and tolerability of cryotherapy. The total FACT-Taxane score was calculated based on an evaluation of skin and nail toxicity. A decrease in the FACT-Taxane score reflects a worsening of dermatological symptoms including skin and nail changes. These endpoints were evaluated at baseline, completion of 3, 6, 9, and 12 weekly doses of paclitaxel. In cases that discontinued 12 weekly doses paclitaxel, neuropathy was evaluated at the discontinuation of the paclitaxel treatment.

Compliance to cryotherapy was determined as good when frozen gloves/socks were appropriately installed during each paclitaxel treatment (total 90 min). In cases who could not wear frozen gloves/socks for continuous 90 min and/or interrupted cryotherapy by wearing thermal gloves/socks inside the frozen gloves/socks, compliance to cryotherapy was determined as poor compliance. Localized skin symptoms of eczema and frostbite and general symptoms of bodily chills and headache were assessed as safety markers of cryotherapy.

### Statistical analysis

The primary outcome was the percentage of patients with a significant decrease in FACT-NTX score according to the intention-to-treat principle. We included all patients who underwent at least one dose of weekly paclitaxel in the efficacy analyses. Age (younger than 65 vs. 66 and older) and CTCAE peripheral neuropathy (grade 0 vs. grade 1) were used as stratification factors for randomization.

In a phase II self-controlled trial of compression therapy comparing the protected hand with the non-protected hand in the same patient, the incidence of CTCAE grade 2 or higher peripheral neuropathy was 21% on the affected hand and 76% on the control hand in breast cancer patients who received nab-paclitaxel treatment [26]. We assumed that the preventive effect of cryotherapy is as effective as compression therapy. Therefore, the percentage of patients with a significant decrease in their FACT-NTX score was estimated at 30 and 70% for the cryotherapy group and control group, respectively. In order to detect the difference between the two groups with 80% power and a significance level for a one-sided test of 0.05, 40 cases are required. Furthermore, taking into account dropout cases, 44 cases (22 cases per group) were planned as the total sample size in this study.

A chi-square test was used as a significance test to compare the percentage of patients with a significant decrease in their FACT-NTX score between the cryotherapy group and control group. A two-sided *p* value < 0.05 was considered significant.

### Findings

#### Patient characteristics

Between February 2017 and September 2018, 44 patients were randomized into the cryotherapy group (*n* = 22) and control group (*n* = 22) (Table 1). All patients were administered at least one dose of weekly paclitaxel. There were no significant differences in age, body mass index, performance status, history of chemotherapy, therapeutic purpose, and weekly paclitaxel treatment regimen between the two groups. Chemotherapy discontinuation occurred in two patients in the cryotherapy group and in three patients in the control group. The reasons for discontinuation were interstitial pneumonia (one patient in each group), worsening peripheral neuropathy (one patients in control group), rash (one patient in cryotherapy group), and depression (one patients in control group).

**Table 1:** Clinicopathological characteristics of the patients

Characteristics	Control group (*n* = 22)		Cryotherapy group (*n* = 22)	
	No.	%	No.	%
Age (years)				
< 77	16	73	17	23
≥ 66	6	27	5	27
Mean body mass index (kg/m^2^ (SD))	1.5 (0.1)		1.5 (0.1)	
Peripheral neuropathy				
CTCAE 0	20	91	20	91
CTCAE 1	2	9	2	9
Performance status				
0	22	100	20	91
1	0	0	2	9
Prior chemotherapy				
None	5	23	4	18
Anthracycline	16	73	18	82
Taxane	1	5	0	0
Treatment				
Neoadjuvant	6	27	4	18
Adjuvant	15	68	17	77
Palliative	1	5	1	5
Paclitaxel regimen				
Paclitaxel alone	9	41	9	41
Paclitaxel and anti-HER2 therapy	13	59	12	55
Paclitaxel and bevacizumab	0	0	1	5

Abbreviations: *CTCAE*, Common Terminology Criteria for Adverse Events; *SD*, standard deviation

### Safety and compliance with cryotherapy

Of the 22 patients in the cryotherapy group, 15 (68%) showed good compliance, while 7 (32%) showed poor compliance. Among subjects showing poor compliance with cryotherapy, four underwent intermittent cryotherapy and three wore thermal insulation gloves/socks inside the frozen gloves/socks. There were no serious side effects associated with cryotherapy, including localized eczema, frostbite, chills or headaches, were observed.

### Efficacy of cryotherapy

**Fig. 1:**
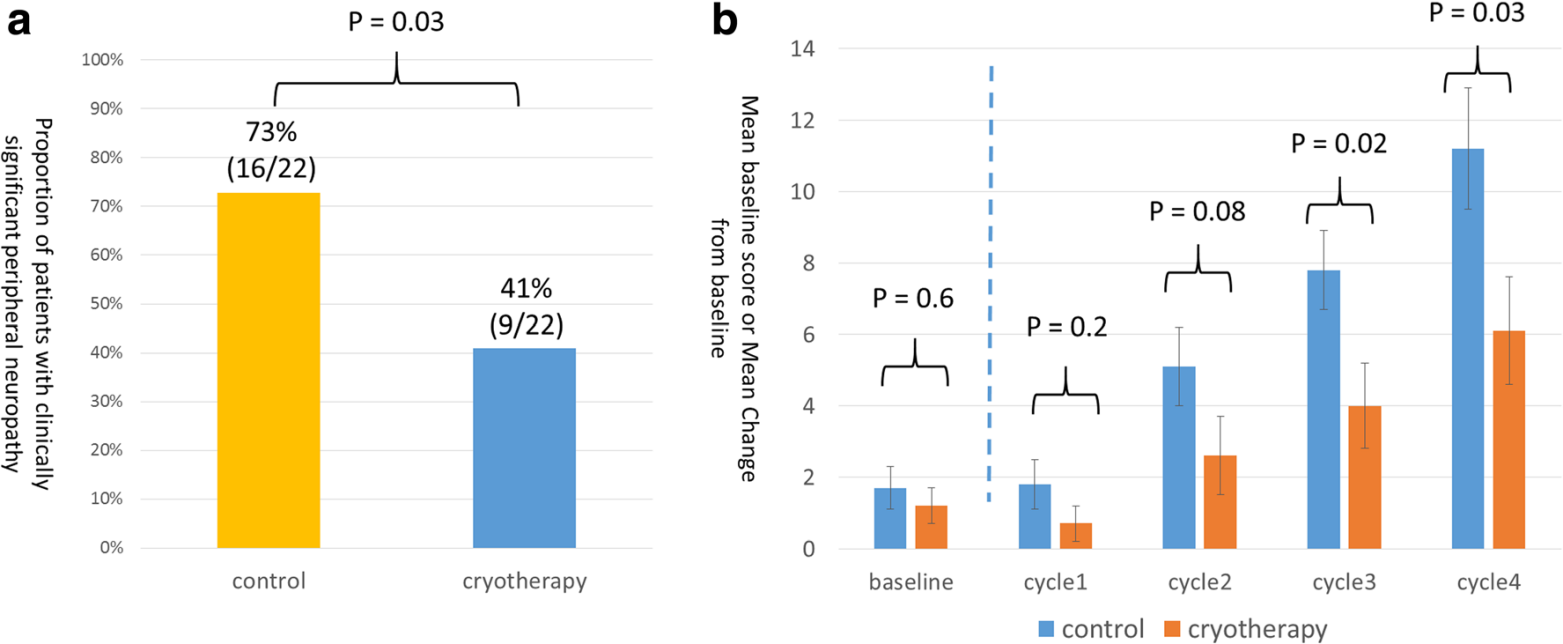
**a** Proportion of patients with clinically significant peripheral neuropathy (i.e., a decrease of 10% or more than 6 points compared with baseline in FACT-NTX score). **b** Baseline and posttreatment FACT-NTX scores

**Fig. 2:**
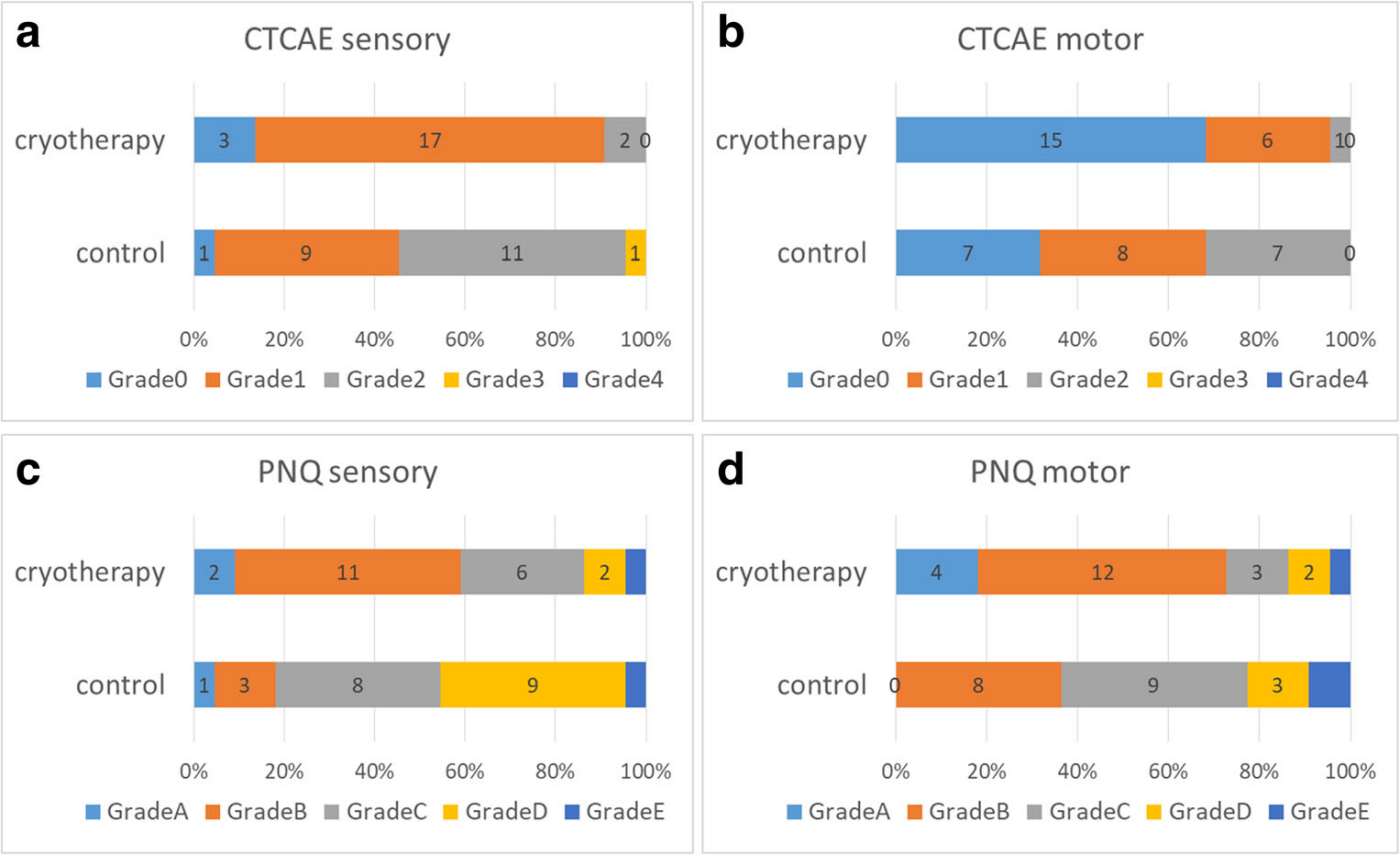
Proportion of worst grades of peripheral neuropathy during paclitaxel therapy, as evaluated using CTCAE version 4.0 and PNQ. **a** CTCAE sensory neuropathy. **b** CTCAE motor neuropathy. **c** PNQ sensory neuropathy. **d** PNQ motor neuropathy. *p* value was evaluated using the Pearson correlation coefficient

**Fig. 3:**
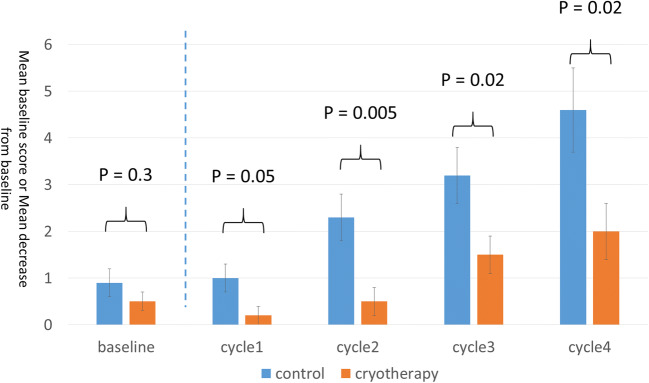
Baseline and posttreatment FACT-Taxane scores

The percentage of patients who showed a marked decrease in FACT-NTX scores was significantly lower in the cryotherapy group than in the control group: (41 vs. 73%, *p* = 0.03). After completion of 12 weekly doses of paclitaxel or termination of paclitaxel treatment, the average decrease in the FACT-NTX subscale score was 6.1 and 11.2 in the cryotherapy and the control groups, respectively, and the magnitude of decrease in the FACT-NTX subscale score was significantly lower in the cryotherapy group (95% CI, 0.5 to 9.6; *p* = 0.03; Fig. 1b). Of the 22 patients in the cryotherapy group in this study, 7 (32%) were determined as poor in compliance to cryotherapy. The incidence of significant peripheral neuropathy as determined using the FACT-NTX tended to be lower in the good compliance group (33%) than in the poor compliance group (57%). The incidence of CTCAE grade ≥ 2 or higher peripheral sensory neuropathy was significantly lower in the cryotherapy group than in the control group (9 vs. 54%; *p* = 0.001; Fig. 2a). The incidence of CTCAE grade ≥ 2 motor peripheral neuropathy was significantly lower in the cryotherapy group than in the control group (5 vs. 32%; *p* = 0.01; Fig. 2b). The incidence of PNQ Grade D or higher peripheral sensory neuropathy was significantly lower in the cryotherapy group than in the control group (14 vs. 42%; *p* = 0.02; Fig. 2c). Meanwhile, there were no significant differences in the incidence of PNQ grade D or higher peripheral motor neuropathy between the cryotherapy and the control groups (14 and 23%; *p* = 0.4; Fig. 2d). The average FACT-Taxane score reduced by 2.0 and 4.6 points in the cryotherapy and the control groups, respectively, with the change being lower in the cryotherapy group (95% CI, 0.4 to 4.8; *p* = 0.02; Fig. 3).

## Discussion

In this study, we investigated the efficacy of cryotherapy for preventing peripheral sensory neuropathy and dermatological adverse events associated with weekly paclitaxel in breast cancer patients. No severe cryotherapy-related adverse events were noted, and the incidence of peripheral neuropathy was lower in the cryotherapy group. These findings indicate that cryotherapy is a well-tolerated and effective modality against peripheral sensory neuropathy and dermatological adverse events in patients treated with weekly paclitaxel.

The clinical practice guidelines of American Society of Clinical Oncology dose not present an established preventive procedure and management protocol for chemotherapy-induced peripheral neuropathy (CIPN) [11]. The use of tricyclic antidepressants, anticonvulsants, various vitamins, or glutamine preparations has been investigated in randomized clinical trials, but none have been proven effective for prevention or alleviation of CIPN [11]. Only duloxetine, a selective serotonin-norepinephrine reuptake inhibitor, has been reported to be effective in alleviating the symptoms of CIPN, but subgroup analysis of chemotherapy regimens have shown no benefit for paclitaxel therapy-induced peripheral neuropathy [22]. Further, duloxetine yields adverse events such as sleepiness and thirst and is limited by low tolerability. There is also no medication that has been established for preventing or treating dermatological adverse events. While external medication may not be helpful, topical steroids, moisturizer or protective varnish is generally administered as a palliative treatment.

Recently, preliminary reports showed the possibility of cryotherapy or compression therapy on affected hands and feet for the prevention of taxane-induced peripheral neuropathy and dermatologic toxicity [4, 8, 18, 19, 26]. In a self-controlled study evaluating the effect of frozen gloves/socks to prevent weekly paclitaxel related peripheral neuropathy in patients with breast cancer, there was a significant decrease in the occurrence of both objective and subjective signs of CIPN [8]. The primary end point in this study was incidence of objective CIPN evaluated by tactile sensation as assessed by the Semmes-Weinstein monofilament test at a cumulative paclitaxel dose of 960 mg/m^2^. While the incidence of objective CIPN events was 81 and 64 on control-sided hands and feet, respectively, 28 and 25% were reported to have had objective CIPN events on the intervention sided hands and feet, respectively. Similarly, in a self-controlled study evaluating the effect of compression gloves to prevent CIPN in breast cancer patients treated with triweekly nab-paclitaxel treatment, less frequent peripheral neuropathy as measured by the CTCAE and PNQ was seen in the treated hand compared with the other control hand [26]. However, these results are still preliminary due to the nature of the study design, and confirmation of the effect in a randomized controlled trial or larger studies is warranted to establish cryotherapy as a preventive procedure for taxane-induced peripheral adverse events. With these findings, we conducted a randomized phase II study to confirm the effect of cryotherapy for the prevention of peripheral neuropathy and dermatological adverse events in breast cancer patients treated with weekly paclitaxel.

Consistent with previous studies, we confirmed the effect of cryotherapy for prevention of taxane-induced peripheral neuropathy and dermatological adverse events. The incidence of clinical meaningful peripheral neuropathy was significantly lower in the cryotherapy group compared with the control group. In this study, peripheral neuropathy was assessed by multiple subjective evaluation methods including FACT-NTX, PNQ, and CTCAE. Among these methods, we determined a change of FACT-NTX score as a primary endpoint because the 11-item neurotoxicity components of the questionnaire were shown to demonstrate excellent consistency and validity with CIPN symptoms and QOL deterioration [3]. Although CTCAE is generally assessed for evaluation of CIPN, physicians are likely to underestimate CIPN symptoms in comparison with patients’ assessment [20]. Thus, CTCAE for peripheral neuropathy was determined as the secondary endpoint in this study. As expected, the percentage of patients with a marked decrease in FACT-NTX scores was significantly lower in the cryotherapy group than in the control group (41 vs. 73%, *p* = 0.03). However, the incidence of peripheral neuropathy in the cryotherapy group is a slightly higher than the incidence reported in previous study [6]. This difference may be attributed to the variation in the chemotherapy regimen. The previous study evaluated 2-week paclitaxel and 2-week docetaxel regimens as well as weekly paclitaxel, which may cause differences in the incidence of peripheral neuropathy. In common, 2-week paclitaxel and docetaxel regimens resulted in less neurotoxicity than 3-week paclitaxel regimens. In the cryotherapy group, the incidence of CTCAE grade ≥ 2 and PNQ grade D or higher was substantially inhibited with rates of 8 and 14%, respectively. These rates are equivalent with the rates reported in previous studies [8, 26]. These multiple evaluations for peripheral neuropathy strengthen the preventive effect of cryotherapy for taxane-induced CIPN. In addition to peripheral neuropathy, we confirmed the efficacy of cryotherapy for preventing dermatological adverse events. In the analysis of the FACT-Taxane score, cryotherapy significantly inhibited hand and foot edema, finger pain, and nails disorders. Dermatological disorders including skin and nail disorder are frequent and cumbersome adverse events in patients undergoing taxane-based chemotherapy. As shown in a previous case-control study, a frozen glove is effective for prevention of docetaxel-induced onycholysis and cutaneous toxicity [18]. Cryotherapy is also an effective prevention procedure for taxane-induced skin toxicity and nail disorders. With these findings, cryotherapy may be an effective procedure in prevention of peripheral dermatologic adverse events to improve compliance with taxane-based chemotherapy. While no serious adverse events related to cryotherapy were observed in this study, moderate cases showed poor compliance with cryotherapy. In patients who showed poor compliance to cryotherapy, there was tendency toward higher incidence of significant peripheral neuropathy compared with patients showing good compliance to cryotherapy. In one study investigating cryotherapy for prevention of docetaxel induced dermatological toxicity, up to 60% of patients discontinued the cryotherapy due to discomfort with the treatment [15]. Patient education and encouragement of adhesion to cryotherapy may be crucial to maximize the protective effect in terms of taxane-induced peripheral toxicity.

There are several limitations to this study. First, this study is a relatively small-sized phase II trial. Our findings about the effectiveness and safety of cryotherapy need to be confirmed in a large clinical trial with objective assessment of peripheral neuropathy, like electromyography. Second, our study focused on weekly-paclitaxel based regimens. Other taxane-based regimens, such as docetaxel or nab-paclitaxel, should be evaluated in future studies. Finally, there was a progressive deterioration in FACT-NTX score in association with the cumulative doses of paclitaxel in the cryotherapy group. Therefore, other modalities, such as compression therapy, may be considered in combination with cryotherapy to prevent CIPN in advanced/recurrent breast cancer where long-term drug administration is required.

## Conclusion

In conclusion, cryotherapy was effective in preventing peripheral neuropathy and dermatological adverse events associated with weekly paclitaxel regimens in breast cancer treatment. Further studies on cryotherapy are warranted to establish a preventive approach for peripheral neuropathy and dermatological adverse events in breast cancer patients undergoing taxane-based chemotherapy.

## Abbreviations

FACT-NTX: Functional Assessment of Cancer Therapy-Neurotoxicity
PNQ: Patient Neurotoxicity Questionnaire
CTCAE: Common Terminology Criteria for Adverse Event
QOL: Quality of life
PS: Performance status
HER2: Human epidermal growth receptor

## References

[1] Alberti P, Rossi E, Cornblath DR, Merkies IS, Postma TJ, Frigeni B, Bruna J, Velasco R, Argyriou AA, Kalofonos HP, Psimaras D, Ricard D, Pace A, Galie E, Briani C, Dalla Torre C, Faber CG, Lalisang RI, Boogerd W, Brandsma D, Koeppen S, Hense J, Storey D, Kerrigan S, Schenone A, Fabbri S, Valsecchi MG, Cavaletti G, Group CI-P (2014). Physician-assessed and patient-reported outcome measures in chemotherapy-induced sensory peripheral neurotoxicity: two sides of the same coin. Ann Oncol.

[2] Capriotti K, Capriotti JA, Lessin S, Wu S, Goldfarb S, Belum VR, Lacouture ME (2015). The risk of nail changes with taxane chemotherapy: a systematic review of the literature and meta-analysis. Br J Dermatol.

[3] Cella D, Peterman A, Hudgens S, Webster K, Socinski MA (2003). Measuring the side effects of taxane therapy in oncology: the functional assesment of cancer therapy-taxane (FACT-Taxane). Cancer.

[4] Eckhoff L, Knoop AS, Jensen MB, Ejlertsen B, Ewertz M (2013). Risk of docetaxel-induced peripheral neuropathy among 1,725 Danish patients with early stage breast cancer. Breast Cancer Res Treat.

[5] Eisenhauer EA, ten Bokkel Huinink WW, Swenerton KD, Gianni L, Myles J, van der Burg ME, Kerr I, Vermorken JB, Buser K, Colombo N (1994). European-Canadian randomized trial of paclitaxel in relapsed ovarian cancer: high-dose versus low-dose and long versus short infusion. J Clin Oncol Off J Am Soc Clin Oncol.

[6] Greenlee H, Hershman DL, Shi Z, Kwan ML, Ergas IJ, Roh JM, Kushi LH (2016) BMI, lifestyle factors and taxane-induced neuropathy in breast cancer patients: the pathways study. J Natl Cancer Inst 109:2 10.1093/jnci/djw206 PMC6093415 27794123

[7] Grisold W, Cavaletti G, Windebank AJ (2012). Peripheral neuropathies from chemotherapeutics and targeted agents: diagnosis, treatment, and prevention. Neuro-oncology.

[8] Hanai A, Ishiguro H, Sozu T, Tsuda M, Yano I, Nakagawa T, Imai S, Hamabe Y, Toi M, Arai H, Tsuboyama T (2018). Effects of cryotherapy on objective and subjective symptoms of paclitaxel-induced neuropathy: prospective self-controlled trial. J Natl Cancer Inst.

[9] Hershman DL, Weimer LH, Wang A, Kranwinkel G, Brafman L, Fuentes D, Awad D, Crew KD (2011). Association between patient reported outcomes and quantitative sensory tests for measuring long-term neurotoxicity in breast cancer survivors treated with adjuvant paclitaxel chemotherapy. Breast Cancer Res Treat.

[10] Hershman DL, Unger JM, Crew KD, Minasian LM, Awad D, Moinpour CM, Hansen L, Lew DL, Greenlee H, Fehrenbacher L, Wade JL, Wong SF, Hortobagyi GN, Meyskens FL, Albain KS (2013). Randomized double-blind placebo-controlled trial of acetyl-L-carnitine for the prevention of taxane-induced neuropathy in women undergoing adjuvant breast cancer therapy. J Clin Oncol Off J Am Soc Clin Oncol.

[11] Hershman DL, Lacchetti C, Dworkin RH, Lavoie Smith EM, Bleeker J, Cavaletti G, Chauhan C, Gavin P, Lavino A, Lustberg MB, Paice J, Schneider B, Smith ML, Smith T, Terstriep S, Wagner-Johnston N, Bak K, Loprinzi CL, American Society of Clinical O (2014). Prevention and management of chemotherapy-induced peripheral neuropathy in survivors of adult cancers: American Society of Clinical Oncology clinical practice guideline. J Clin Oncol Off J Am Soc Clin Oncol.

[12] Hussain S, Anderson DN, Salvatti ME, Adamson B, McManus M, Braverman AS (2000). Onycholysis as a complication of systemic chemotherapy: report of five cases associated with prolonged weekly paclitaxel therapy and review of the literature. Cancer.

[13] Kupfer I, Balguerie X, Courville P, Chinet P, Joly P (2003). Scleroderma-like cutaneous lesions induced by paclitaxel: a case study. J Am Acad Dermatol.

[14] Mackay-Wiggan J, Nair KG, Halasz CL (2003). Onycholysis associated with paclitaxel. Cutis.

[15] McCarthy AL, Shaban RZ, Gillespie K, Vick J (2014). Cryotherapy for docetaxel-induced hand and nail toxicity: randomised control trial. Support Care Cancer.

[16] Mols F, Beijers T, Vreugdenhil G, van de Poll-Franse L (2014). Chemotherapy-induced peripheral neuropathy and its association with quality of life: a systematic review. Support Care Cancer.

[17] Pedersen JV, Jensen S, Krarup-Hansen A, Riis L (2010). Scleroderma induced by paclitaxel. Acta Oncol.

[18] Scotte F, Tourani JM, Banu E, Peyromaure M, Levy E, Marsan S, Magherini E, Fabre-Guillevin E, Andrieu JM, Oudard S (2005). Multicenter study of a frozen glove to prevent docetaxel-induced onycholysis and cutaneous toxicity of the hand. J Clin Oncol Off J Am Soc Clin Oncol.

[19] Scotte F, Banu E, Medioni J, Levy E, Ebenezer C, Marsan S, Banu A, Tourani JM, Andrieu JM, Oudard S (2008). matched case-control phase 2 study to evaluate the use of a frozen sock to prevent docetaxel-induced onycholysis and cutaneous toxicity of the foot. Cancer.

[20] Shimozuma K, Ohashi Y, Takeuchi A, Aranishi T, Morita S, Kuroi K, Ohsumi S, Makino H, Mukai H, Katsumata N, Sunada Y, Watanabe T, Hausheer FH (2009). Feasibility and validity of the patient neurotoxicity questionnaire during taxane chemotherapy in a phase III randomized trial in patients with breast cancer: N-SAS BC 02. Support Care Cancer.

[21] Sibaud V, Leboeuf NR, Roche H, Belum VR, Gladieff L, Deslandres M, Montastruc M, Eche A, Vigarios E, Dalenc F, Lacouture ME (2016). Dermatological adverse events with taxane chemotherapy. Eur J Dermatol.

[22] Smith EM, Pang H, Cirrincione C, Fleishman S, Paskett ED, Ahles T, Bressler LR, Fadul CE, Knox C, Le-Lindqwister N, Gilman PB, Shapiro CL, Alliance for Clinical Trials in O (2013). Effect of duloxetine on pain, function, and quality of life among patients with chemotherapy-induced painful peripheral neuropathy: a randomized clinical trial. JAMA.

[23] Sparano JA, Wang M, Martino S, Jones V, Perez EA, Saphner T, Wolff AC, Sledge GW, Wood WC, Davidson NE (2008). Weekly paclitaxel in the adjuvant treatment of breast cancer. N Engl J Med.

[24] Sparano JA, Zhao F, Martino S, Ligibel JA, Perez EA, Saphner T, Wolff AC, Sledge GW, Wood WC, Davidson NE (2015). Long-term follow-up of the E1199 phase III trial evaluating the role of taxane and schedule in operable breast cancer. J Clin Oncol Off J Am Soc Clin Oncol.

[25] Tanabe Y, Hashimoto K, Shimizu C, Hirakawa A, Harano K, Yunokawa M, Yonemori K, Katsumata N, Tamura K, Ando M, Kinoshita T, Fujiwara Y (2013). Paclitaxel-induced peripheral neuropathy in patients receiving adjuvant chemotherapy for breast cancer. Int J Clin Oncol.

[26] Tsuyuki S, Senda N, Kanng Y, Yamaguchi A, Yoshibayashi H, Kikawa Y, Katakami N, Kato H, Hashimoto T, Okuno T, Yamauchi A, Inamoto T (2016). Evaluation of the effect of compression therapy using surgical gloves on nanoparticle albumin-bound paclitaxel-induced peripheral neuropathy: a phase II multicenter study by the Kamigata Breast Cancer Study Group. Breast Cancer Res Treat.

